# Computational challenges and opportunities in spatially resolved transcriptomic data analysis

**DOI:** 10.1038/s41467-021-25557-9

**Published:** 2021-09-06

**Authors:** Lyla Atta, Jean Fan

**Affiliations:** 1 Department of Biomedical Engineering, Johns Hopkins University, Baltimore, MD USA; 2 Center for Computational Biology, Whiting School of Engineering, Johns Hopkins University, Baltimore, MD USA; 3 Medical Scientist Training Program, Johns Hopkins University School of Medicine, Baltimore, MD USA; 4 Department of Computer Science, Johns Hopkins University, Baltimore, MD USA

**Keywords:** Transcriptomics, Computational biology and bioinformatics

## Abstract

Spatially resolved transcriptomic data demand new computational analysis methods to derive biological insights. Here, we comment on these associated computational challenges as well as highlight the opportunities for standardized benchmarking metrics and data-sharing infrastructure in spurring innovation moving forward.

Advances in single-cell sequencing technologies have enabled high-throughput transcriptomic profiling for individual cells, allowing the characterization and discovery of transcriptionally distinct cell types and cell states. However, current protocols require dissociating cells from tissues, thereby losing potentially valuable spatial information that may inform how cell types and cell states are organized within tissues and how such organization may ultimately impact phenotype and function^1^. To preserve such spatial information, advances in imaging technologies have enabled high-throughput in situ, targeted transcriptomic profiling of pre-selected RNAs at molecular and single-cell resolution^2^. In addition, technologies based on spatially resolved RNA capture followed by sequencing have enabled non-targeted, genome-wide transcriptional profiling at the 10-100 µm pixel resolution^3^. Though the suitability of each spatial transcriptomics technology in addressing a particular biological question will currently involve balancing the need for experimental throughput versus spatial resolution, with current imaging-based technologies generally offering higher spatial resolution but lower experimental throughput and current sequencing-based technologies generally offering higher experimental throughput but lower spatial resolution, all of these resulting large-scale spatially resolved transcriptomic data demand new computational methods to take advantage of this new spatial information to derive biological insights.

## New methods for new data

Given the nascency of such high-throughput spatially resolved transcriptomic technologies, new computational methods for analyzing the resulting data are still actively being developed. Already, computational methods leveraging Gaussian processes^4^, generalized linear models^5^, and spatial autocorrelation analysis^6^ have been developed to identify genes whose expression exhibits significant spatial variability. Some of these methods can also classify different patterns of spatial variation, such as linear or periodic gene expression^4^, as well as identify spatial features such as gene expression hotspots^7^. Such identification of spatially variable genes can lend insight into position-specific phenotypes as well as developmental and migration gradients. Spatial information can also augment the identification of putative cell–cell communication networks. With single-cell sequencing data, cell–cell communication inference has relied on identifying coordinated expression of known ligand–receptor pairs^8^. Computational methods that leverage the added spatial information from spatially resolved transcriptomic data using graph convolutional neural networks^9^, optimal transport approaches^10^, and spatial cross-correlation analysis^6^ can narrow down candidates to ligand–receptor pairs that are spatially colocalized, potentially indicative of autocrine or paracrine signaling. Furthermore, spatially resolved transcriptomic data with co-registered imaging data present additional sources of heterogeneity, such as morphological variability, which can be used for clustering, as differences in morphology can be a proxy for differences in cell states or other functional phenotypes such as cell cycle position, transformation, or invasiveness. Computational methods that incorporate spatial and morphological information in addition to gene expression information have been applied to further dissect heterogeneity in single-cell populations to identify clusters of single cells that are not only transcriptionally distinct but also morphologically and spatially distinct^11,12^. Although these aforementioned computational methods can be applied to both single-cell resolution and multi-cell pixel-resolution spatially resolved transcriptomic data, interpretation of the resulting trends with multi-cell pixel-resolution data will need to take into consideration potential confounding from pixels containing cells of different cell types.

## Analytical challenges and opportunities

Data from spatially resolved transcriptomic technologies present unique analytical challenges and opportunities. For spatially resolved transcriptomic data from in situ imaging-based technologies, individual identified RNA molecules must be aggregated into cells to achieve single-cell resolution transcriptomic profiling. Therefore, reliable cell segmentation is needed to fully dissect the heterogeneity of single cells in their spatial context, as well as to probe their morphological features and to characterize their intracellular variability. Several cell segmentation pipelines exist and work well with images of cells in culture or fluorescent labeled cells^13,14^. Integrating additional information such as cellular transcriptional composition and prior knowledge of cell type-specific gene expression can further enhance segmentation performance, particularly with crowded but transcriptionally distinct cells^15,16^. However, for cells with more complex morphologies such as neurons, additional computational methods for reliable cell segmentation are still needed. Beyond ensuring more accurate estimation of single-cell gene counts, reliable segmentation opens the door to additional downstream computational methods to incorporate subcellular spatial information. For example, by accurately accounting for the subcellular location of RNA counts, these downstream methods enable the prediction of future cellular transcriptional states by inferring RNA velocity in situ or the characterization of the subcellular spatial heterogeneity of RNAs and its functional impact^17,18^.

Likewise, spatially resolved transcriptomic data from sequencing-based, pixel-resolution, spatially resolved RNA capture technologies present a different set of unique analytical challenges. In particular for technologies with larger pixel sizes, transcripts from multiple cells may be captured in each spatially resolved pixel. As such, each resulting spatially resolved transcriptomic profile may reflect multiple cells of different cell types, thereby hindering the identification of cell-type-specific spatial organizational patterns. To overcome this challenge, several computational methods have been developed to deconvolve cell-type mixtures within each multi-cellular spatially resolved pixel, often by integrating the cell-type-specific transcriptomic profiles derived from a suitable single-cell reference^19–21^ or by applying generative modeling approaches^22^. Although these deconvolution approaches infer the proportional representation of cell types within multi-cellular pixels, additional methods are needed to further dissect the spatial organization of cell types and enable the inference of sub-pixel spatial information.

Still, additional computational methods for analyzing spatially resolved transcriptomic data are needed. Notably, although computational methods have been developed to identify and characterize spatial gene expression patterns, we find that additional methods to systematically characterize and statistically evaluate how such patterns relate to anatomical features of tissues such as blood vessels or organ borders are still needed to understand the relationship between structure and phenotype. Furthermore, current computational methods generally limit spatial analysis to individual tissue sections or multiple contiguous sections from the same sample. To analyze samples collected from different individuals, time points, or perturbations, we anticipate that additional computational methods for aligning to a common coordinate system will be needed to compare, contrast, and characterize differences in spatial gene expression patterns and cellular organization^23^.

## Laying a foundation for the future

As these spatially resolved transcriptomic technologies become more widely adopted, we anticipate that beyond the development of new computational methods for spatially informed data analysis, such computational methods must be implemented and made accessible as robust and usable software. This is needed to ensure that users can apply these technologies and analyze the resulting data effectively and efficiently. We believe the software developed to preprocess and analyze spatially resolved transcriptomic data should therefore adhere to best practices in open-source software development, such as providing adequate documentation of software functionality and maintaining responsive issue tracking. Further, support mechanisms need to be made available to promote and incentivize such adherence. Adherence to such best practices will be especially critical to ensure that these technologies and tools are accessible to researchers with more limited computational expertise. Moreover, as these spatially resolved transcriptomic technologies and protocols are further developed to enable data collection from larger tissue sections with more genes and cells across more samples, analytical algorithms and software implementations that are scalable with respect to runtime and memory will also be critical to ensure that these technologies and tools are accessible to researchers with more limited computational resources.

As current spatially resolved transcriptomic technologies continue to mature, we believe standardized metrics and benchmarks will need to be created, to enable comparisons across these technologies, in particular with regards to detection sensitivity, specificity, and capture efficiency. Such standardized metrics and benchmarks will be important for understanding which technologies may be better suited for specific biological questions such as those that demand detection of lowly expressed genes or single-nucleotide variations. Such standardized metrics and benchmarks will also facilitate the development of computational methods for harmonized analysis of data across multiple technologies. In particular for spatially resolved transcriptomic data from in situ imaging-based technologies, standardized metrics for reporting confidence in spot calling, gene identification, and gene-to-cell assignment remain to be established. We anticipate that such specific standardized metrics will be useful in mitigating error propagation to downstream analyses that may lead to incorrect biological interpretations. For example, errors in spot calling, gene identification, and cell segmentation may lead to inaccurate cellular gene expression counts that result in the misidentification of seemingly new, transcriptionally distinct cell types that are the result of propagated technical errors. One challenge towards establishing a set of standardized metrics for spatially resolved transcriptomic data from in situ imaging-based technologies is related to the current dearth of uniform preprocessing pipelines. Many of the current spatially resolved transcriptomic in situ imaging-based protocols rely on in-house image preprocessing pipelines. Although uniform preprocessing pipelines are being established^24^, further efforts are needed to encourage adoption by enhancing their ease of use, offering comparable features to existing in-house pipelines, while maintaining flexibility across available technological platforms, as well as demonstrating robustness and reproducibility across use cases.

In addition, we find that an accessible and centralized infrastructure is currently still needed for sharing spatially resolved transcriptomic data, in particular from in situ imaging-based technologies. Such an accessible and centralized infrastructure already exists for RNA-sequencing data^25^. Further, it makes readily accessible not only the processed gene counts but also raw sequences, as well as metadata on the machines and organisms used to generate the data and metrics regarding the quality of the data such as base call quality scores. Establishing a similar infrastructure for spatially resolved transcriptomic data from in situ imaging-based technologies may prove to be more complex given the range of protocols and modalities that exist and the sheer size of the raw imaging data as well. However, establishing such an accessible data-sharing infrastructure will be especially important for accelerating the development of computational methods to analyze such spatially resolved transcriptomic data, as it ensures the availability of a wide range of data for method testing and enables the characterization of method performance with respect to data quality. We envision that additional discussion and collaboration from the community will be needed to establish the form of processed data and range of standardized metrics most useful for all invested parties, from those interested in developing new computational methods to those interested in further enhancing the technologies, and those interested in probing deeper into datasets for biological insights.

In conclusion, spatially resolved transcriptomic technologies offer an exciting new way of probing the intricate spatial mechanisms at play within tissue ecosystems. Computational methods are needed to enable the characterization of tissue heterogeneity using the high informational content data obtained from such spatially resolved transcriptomic technologies. Still, there remains a need for targeted perturbation, experimental validation, and investigation of generalizability to validate the insights gained from applying these computational methods. For example, although computational methods have been developed to integrate spatial and morphological information in single-cell clustering, further validation is needed to understand if new cell clusters identified through such integrative approaches represent meaningful functional heterogeneity. Furthermore, investigating the extent to which spatial and morphological characteristics of cells are independent of their gene expression can lend insights into other cell intrinsic and cell extrinsic factors that influence cell phenotype. Likewise, intracellular spatial heterogeneity and its functional consequences remain to be characterized. Ultimately, computational methods for analyzing spatially resolved transcriptomic data offer the potential to identify and characterize the heterogeneity of cells within their spatial contexts and contribute to important fundamental biological insights regarding how tissues are organized in both the healthy and diseased settings.

**Fig. 1:**
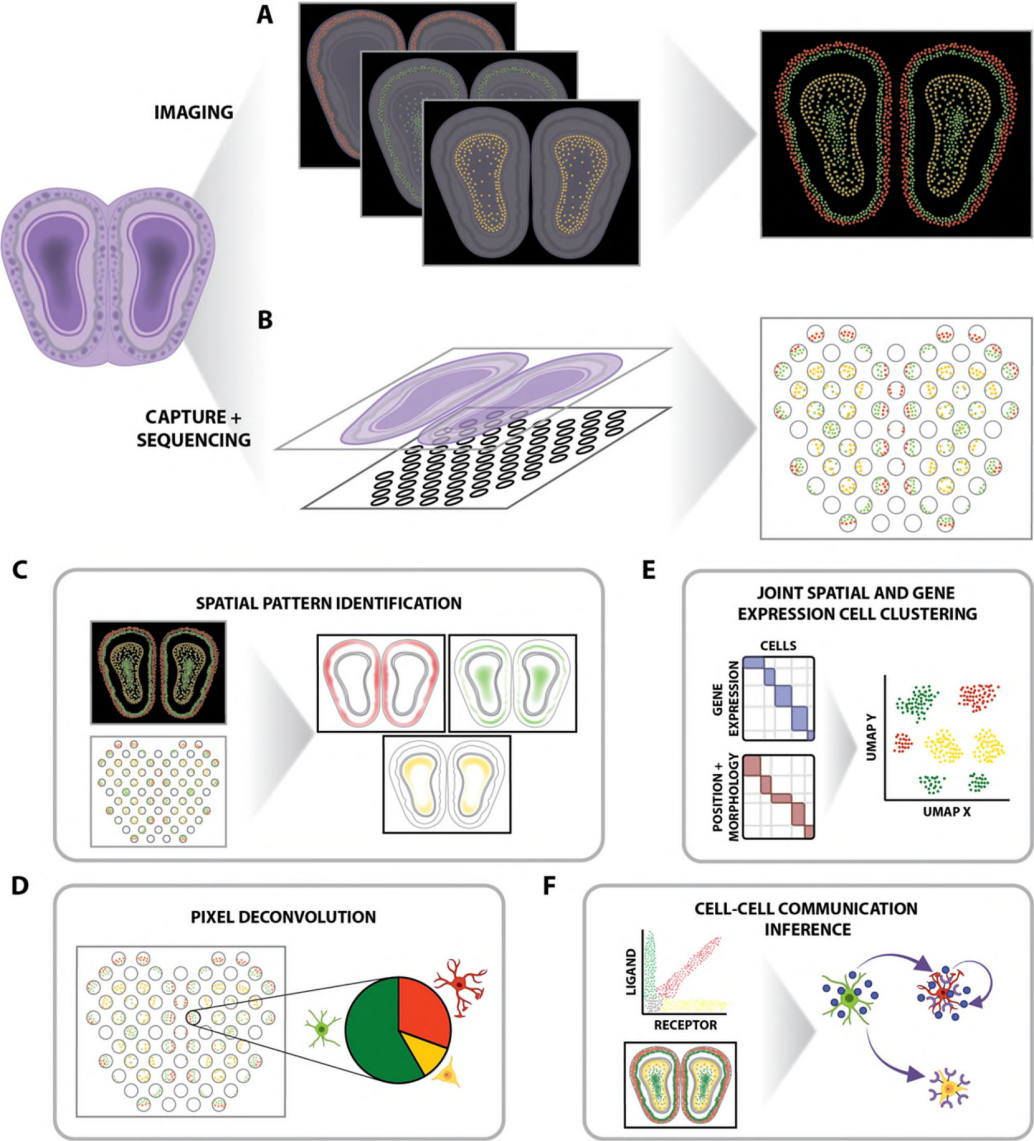
High-throughput spatially resolved transcriptomics data acquisition and analysis. **A** Imaging-based, targeted, in situ transcriptomic profiling at molecular and single-cell resolution or **B** non-targeted, RNA capture, and sequencing at pixel resolution is used to measure RNA in tissues in a spatially resolved manner. Computational methods can be used to **C** identify genes with significantly spatially variable expression patterns, **D** deconvolve multi-cellular pixel-resolution data to determine pixel cell-type composition, **E** combine gene expression, position, and morphological information to cluster cell populations, or **F** identify spatially informed putative cell–cell communication networks.

## References

[1] Maniatis S, Petrescu J, Phatnani H (2021). Spatially resolved transcriptomics and its applications in cancer. Curr. Opin. Genet. Dev..

[2] Zhuang X (2021). Spatially resolved single-cell genomics and transcriptomics by imaging. Nat. Methods.

[3] Larsson L, Frisén J, Lundeberg J (2021). Spatially resolved transcriptomics adds a new dimension to genomics. Nat. Methods.

[4] Svensson V, Teichmann SA, Stegle O (2018). SpatialDE: identification of spatially variable genes. Nat. Methods.

[5] Sun S, Zhu J, Zhou X (2020). Statistical analysis of spatial expression patterns for spatially resolved transcriptomic studies. Nat. Methods.

[6] Miller BF, Bambah-Mukku D, Dulac C, Zhuang X, Fan J (2021). Characterizing spatial gene expression heterogeneity in spatially resolved single-cell transcriptomics data with nonuniform cellular densities. Genome Res..

[7] Edsgärd D, Johnsson P, Sandberg R (2018). Identification of spatial expression trends in single-cell gene expression data. Nat. Methods.

[8] Almet AA, Cang Z, Jin S, Nie Q (2021). The landscape of cell–cell communication through single-cell transcriptomics. Curr. Opin. Syst. Biol..

[9] Yuan Y, Bar-Joseph Z (2020). GCNG: graph convolutional networks for inferring gene interaction from spatial transcriptomics data. Genome Biol..

[10] Cang Z, Nie Q (2020). Inferring spatial and signaling relationships between cells from single cell transcriptomic data. Nat. Commun..

[11] Bae, S., Choi, H. & Lee, D. S. Discovery of molecular features underlying the morphological landscape by integrating spatial transcriptomic data with deep features of tissue images. *Nucleic Acids Res*. 10.1093/nar/gkab095 (2021). 10.1093/nar/gkab095 PMC8191797 33619564

[12] Chidester, B., Zhou, T. & Ma, J. SPICEMIX: integrative single-cell spatial modeling for inferring cell identity. Preprint at *bioRxiv*10.1101/2020.11.29.383067 (2021).

[13] Stringer C, Wang T, Michaelos M, Pachitariu M (2021). Cellpose: a generalist algorithm for cellular segmentation. Nat. Methods.

[14] Greenwald, N. F. et al. Whole-cell segmentation of tissue images with human-level performance using large-scale data annotation and deep learning. Preprint at *bioRxiv*10.1101/2021.03.01.431313 (2021). 10.1038/s41587-021-01094-0 PMC9010346 34795433

[15] Petukhov, V., Soldatov, R. A., Khodosevich, K. & Kharchenko, P. V. Bayesian segmentation of spatially resolved transcriptomics data. Preprint at *bioRxiv*10.1101/2020.10.05.326777 (2020).

[16] Littman R (2021). Joint cell segmentation and cell type annotation for spatial transcriptomics. Mol. Syst. Biol..

[17] Cajigas IJ (2012). The local transcriptome in the synaptic neuropil revealed by deep sequencing and high-resolution imaging. Neuron.

[18] Xia C, Fan J, Emanuel G, Hao J, Zhuang X (2019). Spatial transcriptome profiling by MERFISH reveals subcellular RNA compartmentalization and cell cycle-dependent gene expression. PNAS.

[19] Andersson A (2020). Single-cell and spatial transcriptomics enables probabilistic inference of cell type topography. Commun. Biol..

[20] Cable, D. M. et al. Robust decomposition of cell type mixtures in spatial transcriptomics. *Nat. Biotechnol.* 1–10, 10.1038/s41587-021-00830-w (2021). 10.1038/s41587-021-00830-w PMC8606190 33603203

[21] Elosua-Bayes M, Nieto P, Mereu E, Gut I, Heyn H (2021). SPOTlight: seeded NMF regression to deconvolute spatial transcriptomics spots with single-cell transcriptomes. Nucleic Acids Res..

[22] Miller, B. F., Atta, L., Sahoo, A., Huang, F. & Fan, J. Reference-free cell-type deconvolution of pixel-resolution spatially resolved transcriptomics data. Preprint at *bioRxiv*10.1101/2021.06.15.448381 (2021).

[23] Rood JE (2019). Toward a common coordinate framework for the human body. Cell.

[24] Perkel JM (2019). Starfish enterprise: finding RNA patterns in single cells. Nature.

[25] Edgar R, Domrachev M, Lash AE (2002). Gene Expression Omnibus: NCBI gene expression and hybridization array data repository. Nucleic Acids Res..

